# Medical toolkit organisms and Covid-19

**DOI:** 10.1007/s40656-021-00371-0

**Published:** 2021-02-02

**Authors:** Ulrich E. Stegmann

**Affiliations:** School of Divinity, History and Philosophy, University of Aberdeen, 50-52 College Bounds, Aberdeen, AB24 3DS UK

**Keywords:** Disease models, Non-model organisms, SARS-CoV-2

## Abstract

The Covid-19 pandemic has intensified interest in animals with superior antiviral defences. I argue that the role of such animals in biomedical research contrasts with the role of disease models.

Much biomedical research uses animal models to investigate human diseases. The Covid-19 pandemic shines a spotlight on a very different, and perplexing, way of employing animals in biomedical research: as medical toolkits rather than as models. This note sketches (what I will call) the toolkit strategy for using animals in biomedical research.

As the examples below will show, some animals evolved antidisease strategies that are superior to those of humans. Consequently, some biomedical researchers aim to uncover the underpinning mechanisms and to determine whether the animals’ strategies can be reproduced in humans. In this context, animals are employed like medical toolkits, i.e. collections of ready-to-use equipment and supplies for treating medical conditions. In contrast to disease models, toolkit organisms are investigated *despite and because of the fact* that their features are expected to be *absent* in humans.

Humans and many other mammals defend themselves against viruses by activating the innate immune response (in the first instance). Receptors sense the presence of viral material in the body and trigger pathways that prompt antiviral defences like inflammation. Bats and pangolins are known to cope much better than humans with many viruses, including SARS-Cov-2. As it turns out, they dampen rather than boost their innate immune response. They have permanently inactivated the genes for virus-sensing receptors such as IFIH1 and ZBP1 (Fischer et al. 2020a) and AIM2 (Gorbunova et al. 2020). Furthermore, pangolins have eliminated the cGAS-STING signalling pathway, which receptors trigger when detecting viral material (Fischer et al. 2020b). Bats reduce the inflammation response to TNF-signaling (Gorbunova et al. 2020). Such findings raise the possibility of reproducing these strategies in humans. It may be possible, for instance, to pharmaceutically suppress IFIH1-dependent signaling (Fischer et al. 2020a).

Relatedly, naked mole rats evolved highly effective anticancer strategies (Seluanov et al. 2018). Their cells, for example, quickly cease to proliferate when encountering other cells. Early contact inhibition is due to unusually long versions of hyaluronic acid (HA), which is the main non-protein component in the extracellular matrix. Early contact inhibition may be replicated in humans by lengthening HA (Seluanov et al. 2018). Interestingly, Seluanov and Gorbunova found “a very promising molecule that increases the amount and most importantly the size of HA” in human cell cultures (pers. comm. 5/10/2020).

In the examples above, researchers investigate bats, pangolins, and naked mole rats despite knowing that humans lack their antiviral and anticancer strategies. In fact, researchers investigate these organisms *because* they know this. It would be pointless to probe the strategies’ reproducibility if they already operated in humans. Furthermore, given that the animals’ strategies are expected to be absent in humans, extrapolating the underpinning mechanisms would be unjustified. Thus, employing animals as toolkits for human diseases is not only different from, but contrary to, using them as disease models. Note, however, that the same organism may serve as a toolkit in one context and as a disease model in another.

Green et al. (2018) may be the first HPS discussion of biomedical research into organisms with adaptations that are absent but desirable in humans. The authors construe such organisms as “negative models”, since humans lack their adaptations. They also explore whether it is “possible to generalize or translate” the insights gained, and conclude that the adaptations of some, but not all, such organisms are “applicable or generalizable across species”. Green et al. (2018) glide seamlessly from applicability and translatability to generalizability and therefore miss a key feature of toolkit organisms: whereas the adaptations of these organisms may be applicable or translatable (by introducing or replicating the adaptations in humans), they are not generalisable (because inferences like “since pangolins have mechanism *M*, so do humans” are unwarranted). Put differently, the adaptations may be generalisable in a practical, but never in an epistemic, sense. Toolkit organisms really are distinct from disease models.

Employing animals as medical toolkits is also different from using them as instruments or measuring devices (Germain 2014). When animals serve as instruments, the presence/absence of a state in the animal (e.g. follicular blood spots) indicates the presence/absence of a different state in humans (e.g. pregnancy). This is still an animal-to-human inference, albeit not a generalisation. Yet even such non-generalising inferences are unnecessary and unjustified when animals serve as toolkits.

Note that replicating an organism’s solution in humans need not require replicating the organism’s *mechanism(s)* for that solution. Pangolins dampen the innate immune response against RNA viruses (solution) by inactivating receptor genes (mechanism). In humans, however, inhibiting receptor activity pharmaceutically may achieve the same result. Furthermore, whatever the (dis)similarities between an animal’s mechanism and the one introduced in humans, the latter mechanism may be more or less similar to existing processes in humans. For instance, inhibiting the activity of our receptors would diverge less from our biology than permanently inactivating our receptor genes.

Finally, it is natural to make the uniqueness of the adaptations of toolkit organisms responsible for the failure of extrapolation. But this would be a mistake. Among healthy vertebrates, low-density bones and lack of haemoglobin are unique to Antarctic icefish. But these features are similar to pathological conditions in human anaemias and osteoporosis. Identifying the associated genes in icefish licences inferences about the mutations implicated in human pathologies. Hence, despite their unique adaptations, icefish serve as (“evolutionary mutant”) models for human diseases (Albertson et al. 2009).

This note offered a sketch of biomedical research that employs animals as toolkits. The history of the toolkit strategy, its relation to modelling, and its potential for addressing Covid-19 are future questions for HPS.
